# Supplementary material for: Biochemical Characterization of Parsley Glycosyltransferases Involved in the Biosynthesis of a Flavonoid Glycoside, Apiin
Source: Int J Mol Sci. 2023 Dec 4;24(23):17118. doi: 10.3390/ijms242317118 (PMC10706860; doi:10.3390/ijms242317118)
Supplement: Supplementary file 1 [file ijms-24-17118-s001.zip › ijms-2729790-supplementary.pdf]

**Supplementary Table 1.** Specific primer sequences used in this study.

| PRIMER | SOURCE |
|---|---|
| pColdProS2_PcApiT_F<br>CCATCCATATGGAATCCAAAATGCGTGTC | This paper |
| pColdProS2_PcApiT_R<br>CCATCTCTAGATTATCCCTGGACCACGTA | This paper |
| pET28b_PcGlcT_F<br>GGAATTCCATATGATGGAACTGAATAGTTCAAGCTGC | This paper |
| pET28b_PcGlcT_R<br>CCGCTCGAGTTACTCTTTCCATTTCGTGATCAG | This paper |
| qPCR_PcApiT_F<br>ATCTTCGGTGGTGTTGGTGTGCTT | This paper |
| qPCR_PcApiT_R<br>GGATTCTTCTTTCTCTCTTTGCGG | This paper |
| qPCR_PcGlcT_F<br>CCAGATGCCCCCACTCCT | This paper |
| qPCR_PcGlcT_R<br>TTCTCCGAAAGCCCCCAA | This paper |
| qPCR_ EF-1α_F<br>AGGCTCTTCAGGAGGCTCTTC | [57] |
| qPCR_ EF-1α<br>CAATGTGACAGGTGTGGCAATC | [57] |
| qPCR_ PcFNSI_F<br>GCCTGCTGAGGAAAAACTTG | This paper |
| qPCR_ PcFNSI_R<br>AACTCACGCCAATCCATAGC | This paper |

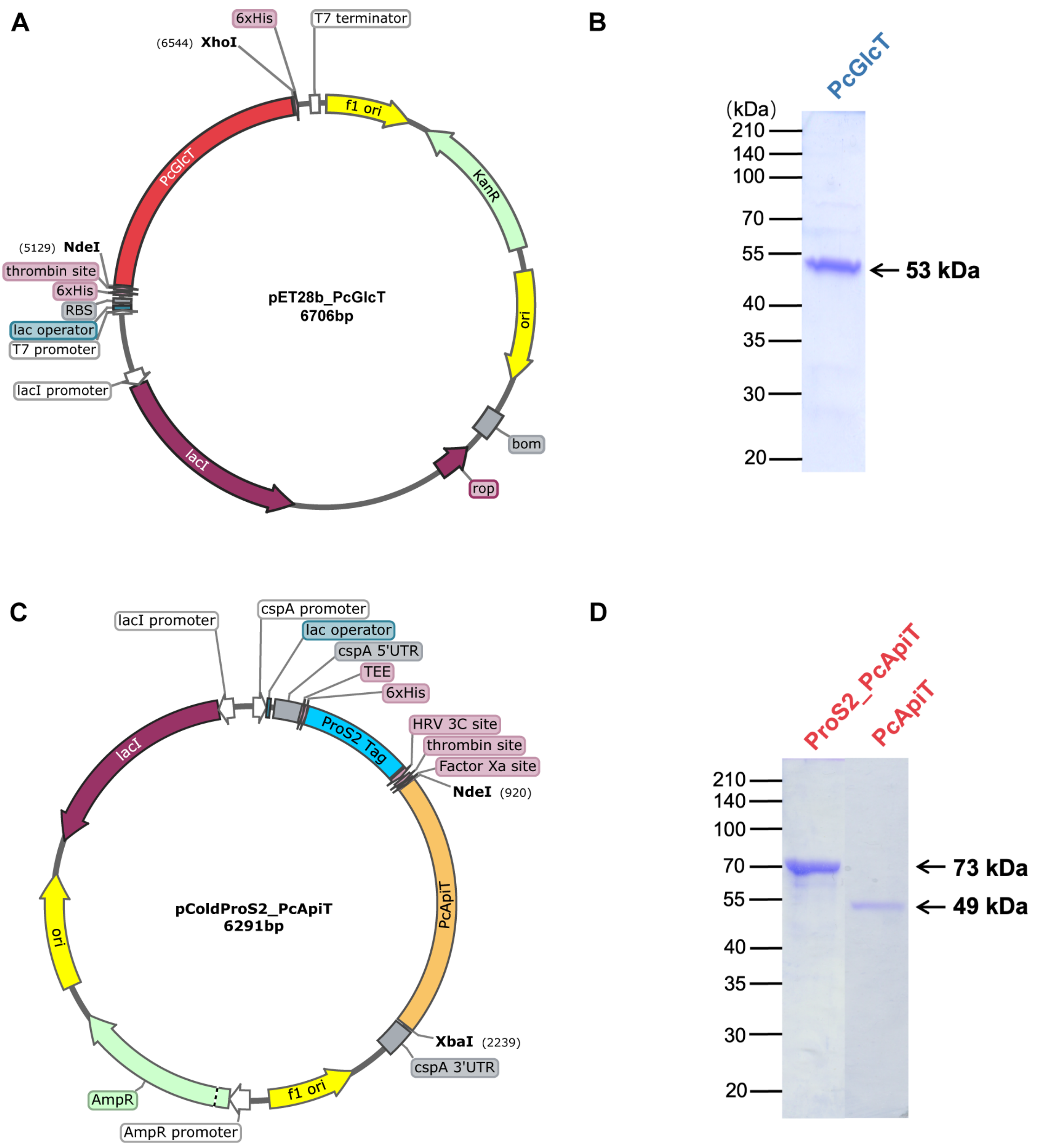


**Supplementary Figure 1:** Vector constructs and the purified recombinant PcGlcT and PcApiT. (A) Schematic diagram of the vector construct for expression of recombinant PcGlcT. (B) SDS–PAGE of the purified recombinant PcGlcT (indicated by an arrow). (C) Schematic diagram of the vector construct for expression of recombinant PcApiT. (D) SDS-PAGE of the purified recombinant PcApiT (indicated by arrows).

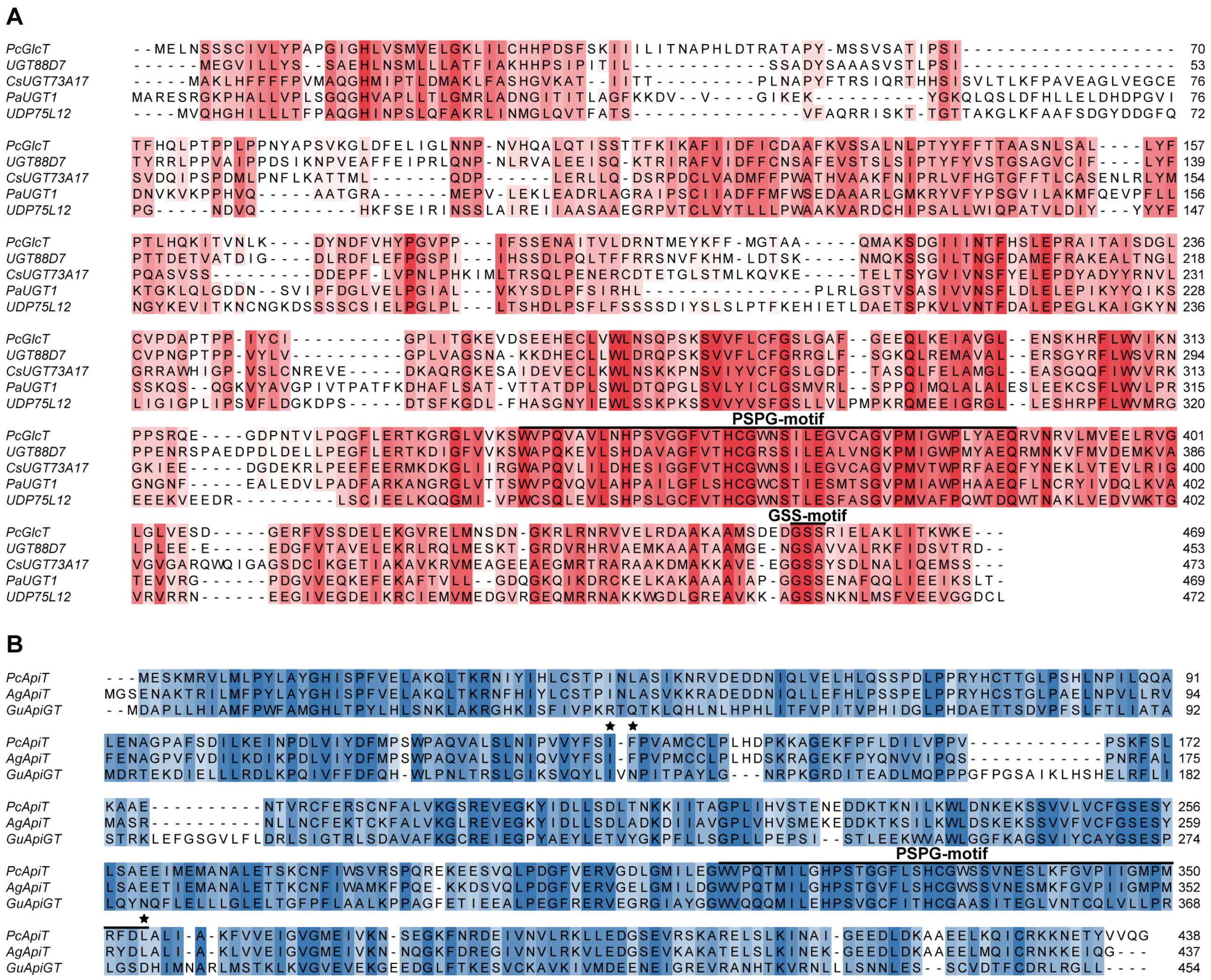


**Supplementary Figure 2.** Multiple sequence alignment of PcGlcT and PcApiT with known related glycosyltransferases. (A) Multiple amino acid sequence alignment of PcGlcT with other flavone glucosyltransferases. These UDP-sugar glycosyltransferases (UGTs) from different UGT families, are glucosyltransferases or exhibit catalytic activity toward apigenin. (B) Amino acid sequence alignment between PcApiT, AgApiT, and GuApiGT. The label section shows the signature PSPG and GSS motifs of plant UGTs. The residues denoted with the star symbol were predicted to be apiose recognition sites for AgApiT.

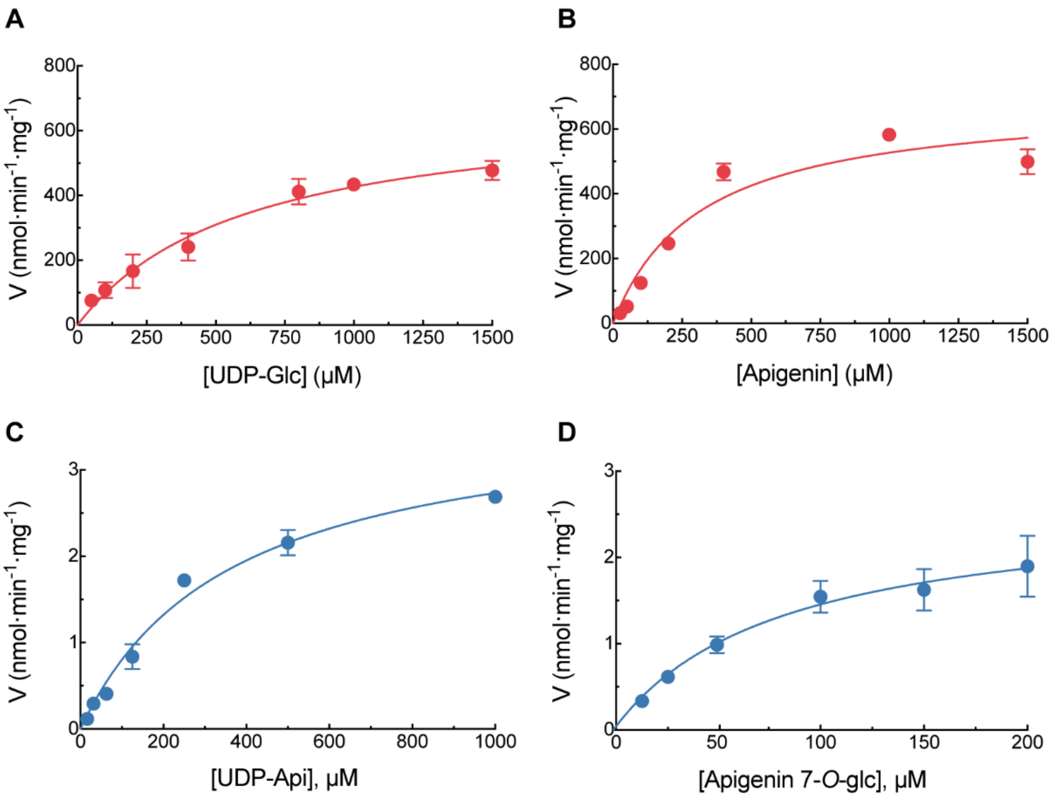


**Supplementary Figure 3:** Kinetic parameters for PcGlcT and PcApiT. (A) Michaelis–Menten plots of PcGlcT for UDP-Glc and (B) apigenin. (C) Michaelis–Menten plots of PcApiT for UDP-Api and (D) apigenin 7-*O*-glucoside. Each bar represents the mean values and standard deviations of three independent samples.
